# Asymmetry in contralateral muscle excitation in proximal vs. distal muscles in upper extremities

**DOI:** 10.3389/fpsyg.2025.1652365

**Published:** 2025-09-22

**Authors:** Alexander Nynes, David McGhie, Morten Andreas Aune, Tore Kristian Aune

**Affiliations:** Department of Sport Science, Sport and Human Movement Science Research Group (SaHMS), Nord University, Levanger, Norway

**Keywords:** asymmetry, contralateral muscle excitation, motor coordination and control, proximal vs. distal, sEMG, bilateral communication

## Abstract

**Introduction:**

The purpose of the study was to investigate potential asymmetry in contralateral muscle excitation (CME) in proximal versus distal muscles. Given the dominant arm’s greater accuracy in unilateral tasks, reinforced by habitual use and neural specialization, along with neurophysiological constraints in the central nervous system and functional differences between proximal and distal muscles, higher CME was hypothesized in both the dominant compared to non-dominant and proximal compared to versus distal muscles. Secondly, a proximal-distal gradient of asymmetry in CME was hypothesized, with more pronounced bilateral asymmetry for distal compared to proximal muscles.

**Methods:**

Isometric shoulder and index finger flexion on the dominant and non-dominant arm was performed at 25, 50, 75, and 100% of maximum isometric force. Muscle excitation was measured using sEMG placed on the non-active contralateral flexor carpi radialis (FCR; distal condition) and on the anterior deltoid (proximal condition) on both the dominant and non-dominant arm.

**Results:**

In the unilateral shoulder flexion (proximal condition), no CME asymmetry between the non-active anterior deltoid on the dominant and non-dominant arm was observed. In contrast, in unilateral index finger flexion (distal condition), a pronounced asymmetry in CME was observed, with the FCR on the dominant arm exhibiting greater CME compared to the FCR on the non-dominant arm.

**Discussion:**

These findings highlight neurophysiological distinctions of the dominant side, especially in distal muscles where refined neural circuits support greater CME. In contrast, the absence of asymmetry in proximal muscles is consistent with their stronger bilateral communication, facilitated by denser interhemispheric and spinal commissural pathways. Overall, the results indicate that CME asymmetries are shaped by both cortical specialization and structural differences in neural connectivity, offering new insight into how dominance and proximal–distal distinctions interact in motor control.

## Introduction

Human movement is characterized by a tendency to favor one side of the body, known as lateral preference. While 25–45% of individuals exhibit a distinct preference for their right foot, this asymmetry is more pronounced in the upper extremities, where up to 90% favor their right arm (Carpes et al., 2010; Čuk et al., 2001; Gilbert and Wysocki, 1992; Porac and Coren, 1977). This preference significantly influences daily motor tasks, with the most evident asymmetry observed in arm function (Aune et al., 2016; Loffing et al., 2024; Mohr et al., 2003). From an environmental perspective, arm function is inherently asymmetrical, as illustrated by everyday tasks like writing or unscrewing a jar, where the non-dominant arm provides stabilization while the dominant arm performs precise, dynamic manipulations (de Poel et al., 2007; Guiard, 1987; Hammond, 2002). As a result, the dominant arm exhibits greater accuracy, dexterity, and efficiency in unilateral motor tasks (Aune et al., 2016; de Boer et al., 2013; Gueugneau and Papaxanthis, 2010; Todor and Kyprie, 1980).

This advantage is attributed to the dominant hemisphere’s more developed motor control areas, strengthened by habitual use and practice, and thus enhancing neural specialization (Changeux and Edelman, 2017; Hammond, 2002; Michel, 2021). Repeated use of the dominant arm refines its neural pathways, improving synaptic connections and enabling faster and more efficient signal transmission (Bruttini et al., 2016; Michel, 2021; Uehara et al., 2014; Vercauteren et al., 2008).

In contrast, the non-dominant arm receives less specialized neural drive to the appropriate muscles, and is even more susceptible to contralateral muscle excitation (CME), where neural signals aimed at specific neural pathway unintentionally interfere with other neural pathways and cause co-activation of muscles (Bruttini et al., 2016).

Subsequently, the inefficient neural circuitry in the non-dominant arm leads to reduced movement precision and making the dominant arm consistently superior in tasks requiring high movement accuracy.

Furthermore, previous research has shown that footedness, rather than dominance, can influence motor unit discharge behavior and force steadiness during dorsiflexion in *tibialis anterior* (Petrovic et al., 2023). In contrast, a later study reported no differences in force variability between dominant and non-dominant limbs during dorsiflexion, but asymmetries appeared at the motor unit level in *tibialis anterior* during less frequently used ankle adduction task, with greater variability and discharge rate in the non-dominant limb (Sahinis et al., 2024).

In addition, it is reported that the biceps brachi in dominant limb receive a higher proportion of coherent synaptic input to motoneurons, reflecting greater and more coordinated spinal motoneuronal output compared to the non-dominant limb (Lecce et al., 2025a). Such enhanced neural drive may contribute to more efficient muscle activation pattern and could influence the degree of contralateral muscle excitation observed between limbs.

These spinal and cortical-level differences raise a broader question that has intrigued researchers for decades: what underlying mechanisms give rise to motor asymmetry in the upper limbs?

Understanding the origin of motor asymmetry has long been a subject of interest, yet its causes remain partially unexplained despite extensive research (Agnew et al., 2004; Corballis, 1998; Hugdahl, 2005; Liederman and Kinsbourne, 1980; McManus, 2002; Parma et al., 2017). Some researchers suggest that the uneven prevalence of right- versus left-arm dominance points to a genetic basis for laterality (Annett, 1978a; Coren and Porac, 1980; Annett, 1978b; Liederman and Kinsbourne, 1980). Conversely, other studies estimate that genetic influence accounts for only 10–20% of side preference, with environmental factors after birth responsible for the remaining 80–90% (Ashton, 1982; Aune et al., 2016; Carpes et al., 2010; Coren, 1993; Harris, 1991; Raymond and Pontier, 2004).

Furthermore, the development of laterality is also shaped by both the structure and function of the nervous system, particularly through bilateral neural communication occurring at multiple levels including the spinal cord, the corticospinal tract, and cortical regions (Delwaide and Pepin, 1991; Hortobágyi et al., 2003; Jankowska et al., 2005a; Kiehn, 2016; Pierrot-Deseilligny and Burke, 2005). At the cortical level, the cerebral hemispheres communicate via transcallosal fibers in the corpus callosum, which enable both inhibitory and excitatory interactions between corresponding cortical areas (Baker, 2011; Kuypers, 1973; Kuypers, 1978; Kuypers, 1964; Rosenzweig et al., 2009). In primates, there is a notably greater density of transcallosal connections between areas in primary somatosensory cortex (S1) and primary motor cortex (M1) associated with proximal muscles compared to those related to distal muscle control (Brodal, 2010; Gould et al., 1986; Jenny, 1979; Pandya and Vignolo, 1971; Rouiller et al., 1994).

On the spinal level, interneurons controlling axial, proximal, and whole-body muscles typically cross the midline to either activate or inhibit motor neurons on the opposite side, whereas those associated with distal muscles exhibit significantly less midline crossing (Jankowska et al., 2005a; Jankowska et al., 2005b). Furthermore, Aune et al. (2016), investigating precision asymmetry, found a proximal-distal gradient in bilateral asymmetry, with more pronounced asymmetry for the index finger compared to the shoulder for both spatial and temporal variables, which was associated to the differences in bilateral communication. Additionally, unpublished findings from our laboratory demonstrate higher contralateral muscle exciation (CME) for proximal compared to distal muscles across different force levels.

Based on these considerations, the present study aimed to investigate the effect of contractions with either the dominant or non-dominant arm on asymmetry in CME in proximal and distal muscles. It was hypothesized that CME (excitation of the non-active muscle) would be higher in the dominant compared to non-dominant proximal and distal muscles. Further, it was hypothesized that there would be a proximal-distal gradient of asymmetry in CME, with more pronounced bilateral asymmetry for distal compared to proximal muscles.

## Materials and methods

### Participants

A sample of 13 healthy university students with no known neurologically disorders, three women (mean age 25.4 ± 7.5) and ten men (mean age 29.4 ± 7.6 years), were recruited. As indicated by the Edinburg Handedness inventory test (Oldfield, 1971), four participants were left-armed (laterality index (LI) = −0.64 ± 0.23) and nine participants were right-armed (LI = 0.87 ± 0.12). All participants provided written, informed consent before the study. The study protocol was reviewed and approved by the Norwegian Agency for Shared Services in Education and Research (SIKT; project number: 152360), and all procedures were conducted in compliance with the latest revision of the Declaration of Helsinki.

### Task

The motor task used in the present study has been extensively described in a previous study (Aune et al., 2013). Briefly explained, participants were positioned in a custom-made chair 2.5 m from a screen (148 × 110 cm), and the task involved pulling a securely mounted S-type push-pull load cell using both maximal voluntary isometric contraction (MVIC) and submaximal contraction in both a proximal (shoulder flexion) and distal (index finger flexion) movement. Participants first completed three MVICs with either elbow or finger flexion. Based on the peak force in the MVICs, the submaximal force (25, 50, and 75%) was calculated, and participants performed three submaximal voluntary contractions at each force level in both the proximal and distal conditions.

### Apparatus

**Figure 1:**
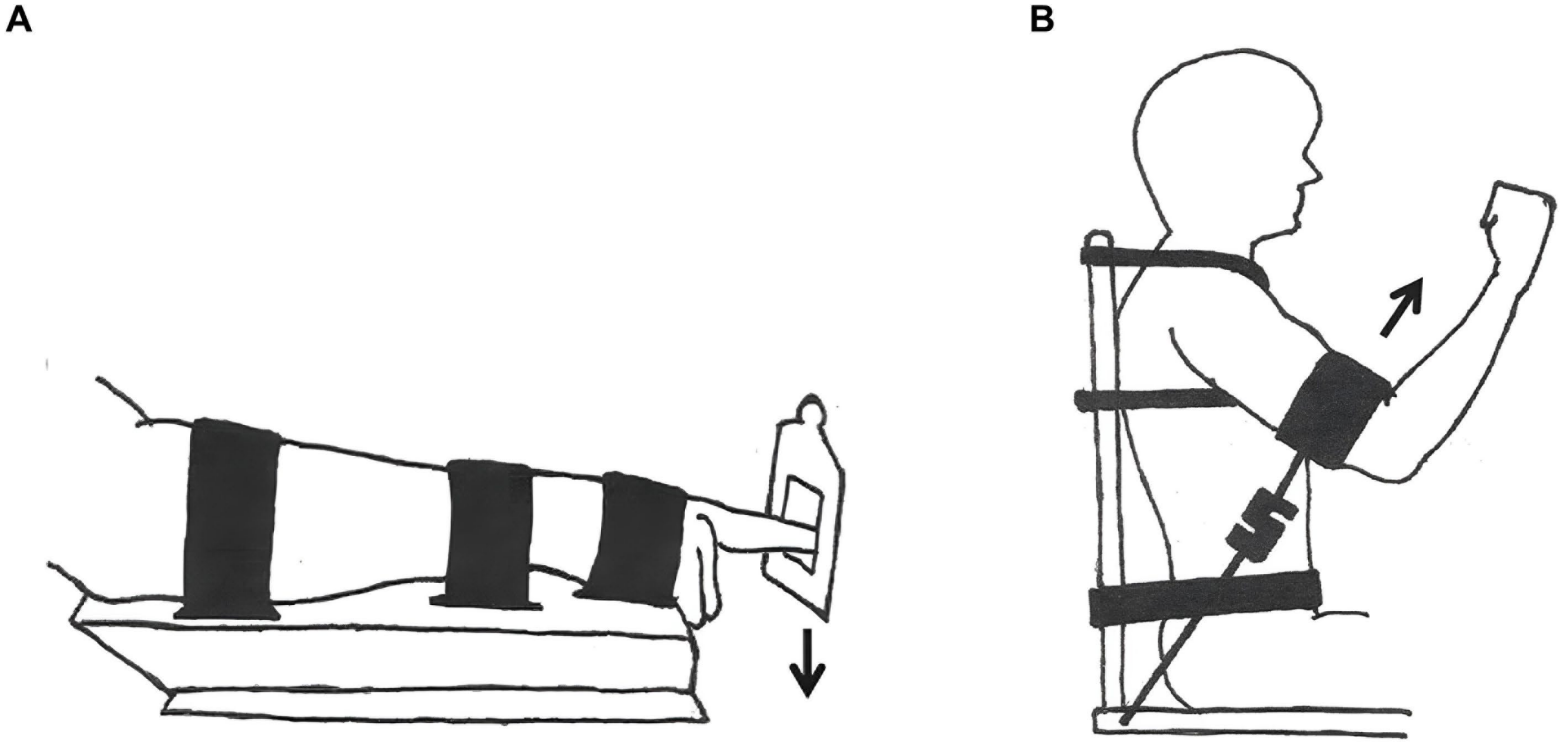
To restrict participant movement and eliminate mechanical, postural, or synergistic muscle involvement, straps and bands were applied. The positioning of these restraints was standardized: for the distal condition **(A)**, a strap was secured 2 cm below the metacarpophalangeal joint of the index finger, while for the proximal condition **(B)**, it was placed on the lower part of the humerus, 5 cm above the elbow joint. sEMG electrodes were affixed to the flexor carpi radialis (FCR) in the distal condition and to the anterior deltoid in the proximal condition. The trunk and upper arm (humerus) were maintained at a 45° angle, with the index finger positioned horizontally. Force transducers were aligned with the direction of the applied force in each condition, as indicated by black arrows.

To ensure isolated unilateral contractions, a custom-made chair and apparatus were developed. Straps and bands were applied to minimize postural instability and restrict movement to a single degree of freedom, allowing targeted muscle activation (Figures 1A,B). For index finger extension, a steel platform combined with straps limited motion exclusively to the intended joint (Figure 1B). During shoulder flexion tasks, additional straps around the waist and chest maintained joint isolation by restricting extraneous movement (Figure 1A).

### Measurements

#### Surface electromyography

To measure surface electromyography (sEMG) activity on both the dominant and non-dominant arm of the participants, recordings were taken from the flexor carpi radialis (FCR) for the distal condition and the anterior deltoid for the proximal condition. To ensure accurate muscle signal acquisition, sEMG electrodes were positioned following SENIAM’s standardized guidelines (Hermens et al., 2000). Before applying the self-adhesive electrodes (Dri-Stick Silver circular sEMG Electrodes AE-131, NeuroDyne Medical, MA, USA), which have an 11 mm contact diameter and a 20 mm center-to-center distance, the participants’ skin was prepared to minimize impedance (Konrad, 2005; Nazmi et al., 2016). To further reduce noise, conductive gel (Signa Gel, Parker Laboratories Inc., Fairfield, NJ, USA) was applied to the electrodes.

The sEMG signals were captured using MuscleLab software (MuscleLab version 10.200.90.5095, Ergotest Technology A/S, Stathelle, Norway), which was also employed for processing the raw sEMG data. Signals underwent amplification and filtering through a preamplifier positioned near the detection site, followed by bandpass filtering (high-pass at 20 Hz, low-pass at 500 Hz). Subsequently, the signals were converted to root mean square (RMS) using a hardware circuit network (frequency response of 450 kHz, averaging constant of 12 ms; total error ± 0.5%) with a common mode rejection ratio of 106 dB. The mean RMS was computed for each muscle during the isometric contractions. To normalize the sEMG signal during index finger and shoulder flexion, participants performed three 6-s isometric contractions (MVIC) targeting the relevant muscles on the dominant and non-dominant arm. The peak sEMG amplitude from these contractions, performed using the same movements as the tasks of interest, was used for normalization (Besomi et al., 2020).

#### Force

Force (in Newtons) was measured with an S-type push-pull load cell transducer (Ergotest Technology A/S), connected to the index finger and elbow via static wires. Data was sampled at 200 Hz during the voluntary contractions. The transducers were aligned with the direction of force exerted, indicated by black arrows in Figure 1. A MuscleLab 6,000 data synchronization unit (DSU) captured the force data, which was then processed using a five-point differential filter in MuscleLab software (version 10.200.90.5095, Ergotest Technology A/S).

### Procedure

Prior to testing, preparation of participants’ skin involving shaving, cleaning with alcohol, and lightly abrading the skin to minimize impedance was done before electrode placement. Participants performed unilateral voluntary contractions using proximal effectors (elbow flexion) and distal effectors (finger flexion) with the dominant and non-dominant arm, with the starting condition counterbalanced among participants During the trials participants were instructed to keep their non-target arm relaxed at their side.

Each experimental condition started with a brief instruction of the task, followed by MVICs and three submaximal contractions at 25, 50, and 75% of MVIC for both proximal and distal muscles in the dominant and non-dominant arm, totaling 48 voluntary contractions. A one-minute rest interval was provided between trials to minimize fatigue. MVIC trials were performed for 6 s, whereas submaximal trials lasted 12 s. These durations were selected to ensure reliable contralateral sEMG recordings. During the contractions, participants received visual feedback on a screen positioned 2.5 m away in front, displaying the target force they needed to achieve at various relative force levels.

### Data analysis

In all experimental conditions, muscle contractions were performed unilaterally, while sEMG amplitudes (converted to RMS) were recorded from the homologous muscles on the side not engaged in the voluntary contractions. Specifically, sEMG amplitudes from the muscles on the non-active arm – whether dominant or non-dominant – were used to identify any CME and served as the basis for further analysis. For MVICs, which lasted 6 s, only the sEMG amplitudes from the 2–5 s time frame were included in the analysis to capture steady-state amplitudes. For submaximal contractions, steady- state amplitudes in the time frame of 2–10 s were analyzed, with the initial and final 2 s of each trial excluded from further analysis to account for potential unwanted influences during the test period’s beginning and end. For example, amplitudes in the first 2 s might reflect adjustments to the task, whereas the last seconds could be affected by muscle fatigue, loss of concentration, or increased effort at the end (Lorås et al., 2012; Moe-Nilssen and Helbostad, 2005; Repp and Penel, 2004).

### Statistical analysis

To compare the submaximal force levels across muscles and arms, one-way repeated ANOVAs were run for each force level, with all combinations of muscles and arms as factors. To determine the presence and degree of asymmetry in CME between the dominant and non-dominant arm in proximal and distal muscles across force levels, two-way repeated ANOVAs were run separately for the proximal and distal muscles, with arm and force level as factors and a two-way interaction. Differences in arm or force level were assessed through *post hoc* tests with a Bonferroni correction for multiple comparisons. No values were considered outliers, determined as 3 SD outside the group mean (combination of muscle, arm, and force level).

A sensitivity power analysis for repeated measures ANOVA was performed *a posteriori* to determine the minimum detectable effect size for each main effect comparison (arm, force level) using G*Power 3.1.9.7 (Faul et al., 2007), given *α* = 0.05, *β* = 0.80, groups = 1, measurements = 2 or 4, *n* = 13, and effect size specification “as in SPSS,” indicating the ability to detect large effects. Effect size was reported as Hedges’ *g*, derived from the differences in estimated marginal means and standard errors, and interpreted according to Cohen (2013) as trivial <0.2, small ≥0.2, moderate ≥0.5, and large ≥0.8. Statistical analysis of the potential difference between the proximal and distal muscles was only deemed necessary if both muscles showed significant asymmetry.

Normality of residuals (Cheng et al., 2010) was assessed visually with histograms and normal-probability plots, as well as through kurtosis and skewness values. For the three one-way ANOVA models, absolute z-scores of both kurtosis (|0.12–0.91|) and skewness (|0.22–0.81|) were <|1.96|. For both two-way ANOVA models, absolute z-scores of kurtosis (|0.92–1.11|) were <|1.96|, whereas z-scores of skewness (|2.57–4.35|) were >|1.96|. The residuals for both models showed a slight right-skew (≤1.03). However, this was not deemed severe enough to preclude interpretation, considering the robustness of repeated measures ANOVA against non-normality with skewness ≤2.31 (Blanca-Mena et al., 2022). Sphericity was assessed with Mauchly’s test, using the Greenhouse–Geisser correction if *ε* < 0.75.

All statistical analyses were performed in SPSS version 29.0.1.1 (IBM Corporation, Armonk, NY, USA). The level of statistical significance was set at *α* = 0.05.

## Results

The actual relative force produced at each submaximal force level in the proximal condition was measured to be 24.3 ± 1.0%, 48.7 ± 1.5%, and 72.4 ± 2.1% in the dominant arm, and 23.8 ± 0.9%, 48.0 ± 2.0%, and 71 ± 3.3% in the non-dominant arm. In the distal condition, the relative force produced was 23.4 ± 2.2%, 47.4 ± 2.7%, and 73.2 ± 2.8% in the dominant arm, and 23.1 ± 2.3%, 48.7 ± 3.0%, and 73.3 ± 3.8% in the non-dominant arm. There were no significant differences in relative force between any combination of muscle and arm at force level 25% (F_2.114,25.365_ = 1.446, *p* = 0.255), 50% (F_1.722,20.660_ = 0.779, *p* = 0.454), or 75% (F_1.980,23.760_ = 1.335, *p* = 0.282).

**Figure 2:**
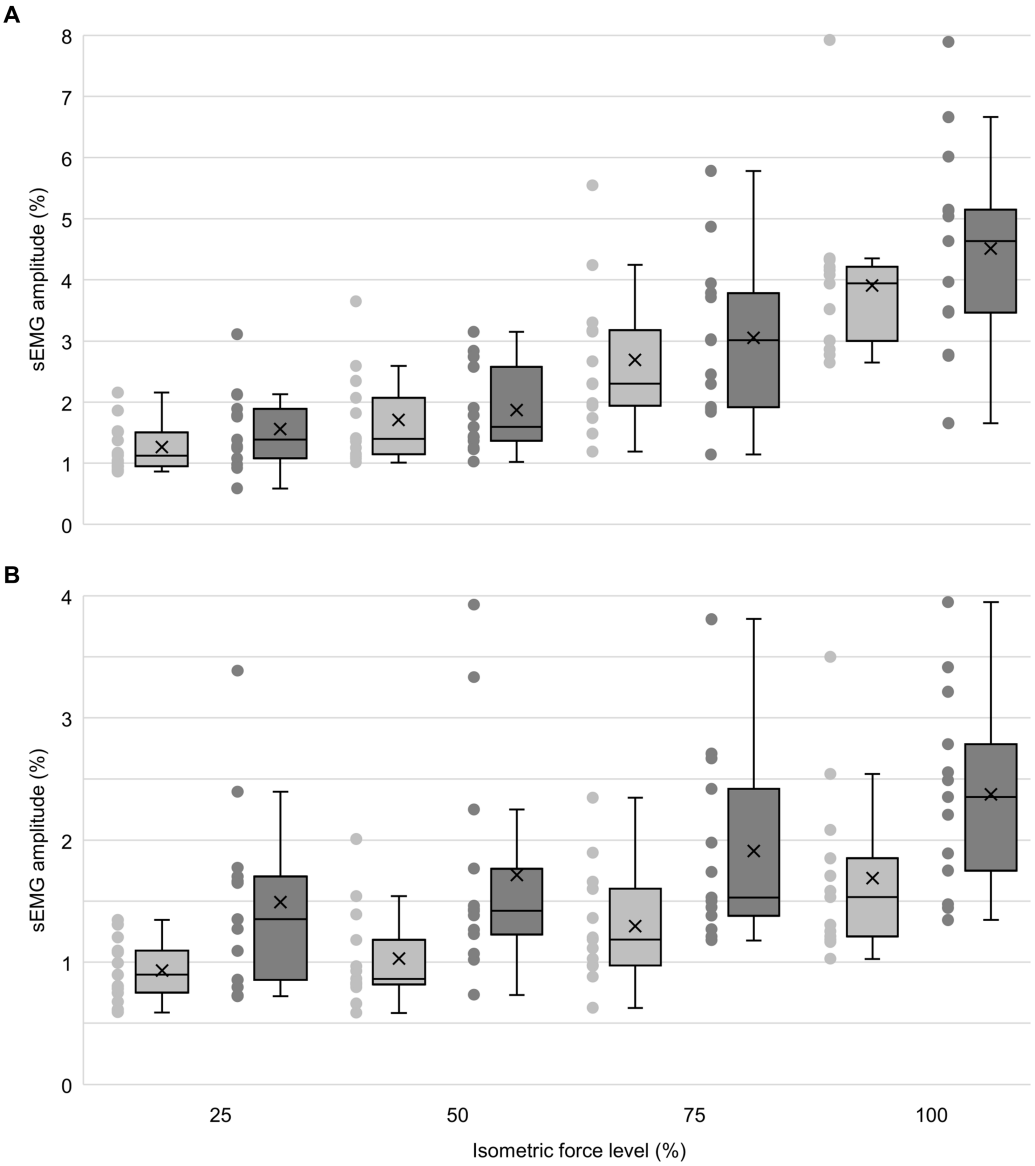
Box plots of normalized sEMG amplitude (%) in proximal and distal muscles on the dominant (dark grey) and non-dominant (light grey) arm across relative force levels (*n* = 13). The horizontal line represents the median and X represents the mean. Quartiles were calculated inclusive the median, and whiskers represent 1.5 x interquartile range outside the first and third quartile. For proximal muscles **(A)**, no significant difference in contralateral muscle excitation was found between the dominant and non-dominant arm. For distal muscles **(B)**, a significant difference in contralateral muscle excitation was found, with the flexor carpi radialis showing higher sEMG amplitude on the dominant arm compared to the non-dominant arm (*p* < 0.05). Note that the *y*-axis scaling differs between panel **(A,B)**.

For CME in the proximal muscle, there was no significant interaction between arm and force level (F_1.566,18.797_ = 1.772, *p* = 0.200; Figure 2A), and no significant difference in CME between the dominant and non-dominant arm (F_1,12_ = 1.977, *p* = 0.185, *g* = 0.37). There was an overall effect of force level (F_3,36_ = 70.830, *p* < 0.001), with *post hoc* tests showing significant increases in CME between all increasing force levels (25–50% *p* = 0.042, *g* = 0.87; 50–75% *p* < 0.001, *g* = 1.41; 75–100% *p* < 0.001, *g* = 1.49; Figure 2A).

Similarly, for the distal muscle, there was no significant interaction between arm and force level (F_1.765,21.182_ = 0.218, *p* = 0.779). However, there was a significant difference in CME between the dominant and non-dominant arm (F_1,12_ = 13.624, *p* = 0.003, *g* = 0.99; Figure 2B), with higher CME in the dominant arm. Further, there was an overall effect of force level (F_3,36_ = 19.557, *p* < 0.001), with post hoc tests showing significant increases in CME between 25 and 100% (*p* < 0.001, *g* = 1.53), 50–100% (*p* = 0.004, *g* = 1.20), and 75–100% (*p* = 0.024, *g* = 0.95), as well as between 25 and 75% (*p* < 001, *g* = 1.64).

## Discussion

The present study investigated potential asymmetries in contralateral excitation between the non-active dominant and non-dominant arms, specifically in proximal (anterior deltoid) and distal (flexor carpi radialis) muscles. Participants performed unilateral isometric contractions with the dominant and non-dominant arm at various force levels, determined by each participant’s individual maximal voluntary contraction. By analyzing the amplitude of surface EMG (sEMG) signals from the proximal and distal muscles of interest on the arm not engaged in the voluntary contractions, the study aimed to identify potential differences in CME between the dominant and non-dominant arm. The main finding was a significant asymmetry in the distal condition, specifically CME was greater in the dominant arm’s distal muscle when the non-dominant arm exerted the force, compared to the reverse. This suggests a functional advantage of the dominant side, potentially linked to enhanced control due to habitual use and more refined neural pathways (Abreu et al., 2015; Cernacek, 1961; Cincotta et al., 2003; Cincotta and Ziemann, 2008; Zijdewind et al., 2006; Zijdewind et al., 1998). Repeated use of the dominant arm likely strengthens reentrant neural circuits in the dominant hemisphere, supporting more precise and isolated activation and potentially suppressing unintended excitation in contralateral muscles (Aune et al., 2016; de Boer et al., 2013; de Poel et al., 2007; Gueugneau and Papaxanthis, 2010; Huber, 1999; Peters and Durding, 1979; Porac and Coren, 1981; Smoll and Schutz, 1978; Todor and Kyprie, 1980; Todor et al., 1982; Truman and Hammond, 1990). This neural specialization supports precise and isolated muscle excitation, which may explain the increased CME observed during non-dominant arm contractions in the distal condition. Functional asymmetry thus contributes to this effect, as the dominant hemisphere’s robust pathways can facilitate focused excitation and suppress unintended excitation in contralateral homologous muscles (Bruttini et al., 2016; Changeux and Edelman, 2017; Hammond, 2002; Michel, 2021).

In contrast, no significant CME asymmetry was observed between proximal muscles on the dominant and non-dominant side. Given proximal muscles frequent involvement in everyday bilateral activities (e.g., lifting, walking, running), these muscles may benefit from more balanced and integrated neural control across hemispheres (Aune et al., 2016; Cratty, 1962). As a result of this consistent bilateral engagement, the neural circuits connecting proximal muscles are often more refined, which could enhance their responsiveness to contralateral excitation and help explain the observed symmetry in CME. As such, a direct comparison of asymmetry between proximal and distal muscles was not warranted based on the current findings. Additionally, the study found that CME increased with higher levels of voluntary force generated (25, 50, 75, and 100%) in both muscle groups, with the effect being more pronounced in proximal muscles. This provides further support for the notion that unilateral motor activity can elicit contralateral neural responses (Abreu et al., 2015; Cernacek, 1961; Cincotta et al., 2003; Cincotta and Ziemann, 2008; Heming et al., 2019; Zijdewind et al., 2006; Zijdewind et al., 1998).

Structural differences in interhemispheric and spinal connectivity further help in explaining the present findings. A key structural distinction is the greater number of commissural fibers in the corpus callosum and interneurons in the spinal cord that connect proximal compared to distal muscles (Matsumoto et al., 2021; Uehara et al., 2014; Vercauteren et al., 2008), enabling stronger bilateral communication and likely contributing to the absence of CME asymmetry in proximal muscles. This aligns with Aune et al. (2017), who reported that training proximal effectors yields greater bilateral transfer of motor learning to homologous effectors compared to training distal effectors, an effect linked to this higher level of bilateral communication. In contrast, distal muscles, with fewer commissural connections, appear to rely more on unilateral cortical control, which may underlie the CME asymmetry observed between dominant and non-dominant limbs.

Neurophysiological research complements this interpretation. Lecce et al. (2025a) showed that, although baseline synaptic noise is similar between limbs, training the non-dominant limb reduces synaptic noise more than training the dominant limb, leading to greater gains in force accuracy. This suggests that less-refined neural circuits have greater adaptive capacity, which may parallel the weaker connectivity of distal muscles and their greater CME asymmetry. In contrast, the robust bilateral circuits of proximal muscles may provide a stable platform for symmetrical activation across motor tasks. Additionally, Lecce et al. (2025a,b) demonstrated that the dominant limb exhibits higher maximal force and EMG amplitude than the non-dominant limb, associated with a greater proportion of coherent synaptic input to motoneurons. Furthermore, our findings of CME asymmetry in distal but not proximal muscles parallels evidence from the lower limb. Petrovic et al. (2023) reported no asymmetry in motor unit behavior during dorsiflexion, whereas Sahinis et al. (2024) found that asymmetries emerged only at the motor unit level during the less habitual task of ankle adduction, where the non-dominant limb showing greater discharge rate and variability. The fact that these asymmetries were restricted to distal control at the motor unit level corresponds closely to our observation of CME asymmetry in distal muscles, reinforcing the view that distal effectors are more susceptible to lateralized differences than proximal muscles.

Although the present study did not directly measure motoneuronal coherence, such asymmetries in neural drive, together with functional and structural connectivity differences, likely contribute to the stronger CME observed in the dominant distal muscle when the non-dominant muscle is active. Complementing this, studies by Farmer et al. (1993) and Johnson et al. (2017) emphasize that a key determinant of motor neuron excitatory threshold is the distribution and coherence of synaptic inputs. Specifically, Differences between dominant and non-dominant limbs in this organization could therefore contribute to CME asymmetries: more coordinated or preferentially distributed inputs in the dominant limb may support stronger and more efficient contralateral recruitment, whereas the non-dominant side, with less refined organization, could have a greater predisposition to crosstalk and variability. This perspective highlights that excitation asymmetries are shaped not only by cortical specialization but also by spinal-level determinants of motoneuron excitability, with the dominant limb benefiting from both more coherent inputs and more favorable thresholds.

### Practical implications

The present findings contribute to a deeper understanding of how muscle group and limb dominance shape contralateral muscle excitation. Taken together, the results indicate that habitual patterns and underlying neurophysiological distinctions jointly influence the organization of motor control, thereby providing new perspectives on the neural mechanisms underlying asymmetry. For distal muscles the observed CME asymmetry highlights the role of functional asymmetry in neuromuscular activation, emphasizing the need to consider these differences in interventions aimed at improving fine motor control and dexterity (Green and Gabriel, 2018; Šarabon et al., 2020). In contrast, the limit or absence of CME asymmetry in proximal muscles reflect more balanced bilateral communication patterns, which indicate that these muscles probably respond more uniformly to unilateral training. This supports the idea that proximal muscles are particularly receptive to interlimb transfer effects, where training one limb facilitates functional improvements in the contralateral limb (Aune et al., 2017; Swift, 1903). Such interlimb transfer effects hold relevance for rehabilitation protocols, including for example post-stroke therapy, where enhancing bilateral coordination is essential for recovery. The present findings also inform what could be likely expectations with regard to progress in training and rehabilitation. For proximal muscles, which are characterized by strong bilateral communication, relatively greater interlimb transfer may be expected, since training of one limb is likely to facilitate gains in the opposite limb as well (Aune et al., 2017). In contrast, distal muscles demonstrate clearer dominance-related asymmetry and weaker transfer, suggesting that progress in the non-dominant side may be slower and require more specific and sustained practice.

## Conclusion

The most prominent finding in the present study is a clear CME asymmetry in distal muscles, where the dominant arm exhibits greater excitation during non-dominant arm activation. This asymmetry likely reflects the dominant limbs more refined and coherent neural circuits, shaped through habitual and frequent use supported by cortical specialization as well as structural differences in neural connectivity. In contrast, the absence of significant CME asymmetry in proximal muscles reflects enhanced bilateral communication mediated by denser interhemispheric and spinal commissural pathways and is in accordance with the habitual and frequent use of bimanual coordination of proximal muscles in everyday movements. Additionally, the study found that CME increased with higher levels of voluntary force output in both muscle groups, with a more pronounced effect observed in proximal muscles. These findings underline the importance of considering muscle group and limb dominance in both training and rehabilitation protocols. By acknowledging habitual and neurophysiological distinctions, future interventions can be better designed to optimize motor training in general, and for rehabilitation and recovery training in particular.

## Data Availability

The raw data supporting the conclusions of this article will be made available by the authors, without undue reservation.

## References

[ref1] Abreu R. Lopes A. A. Sousa A. S. Pereira S. Castro M. P. (2015). Force irradiation effects during upper limb diagonal exercises on contralateral muscle activation. J. Electromyogr. Kinesiol. 25, 292–297. doi: 10.1016/j.jelekin.2014.12.004, PMID: 25592384

[ref2] Agnew J. A. Zeffiro T. A. Eden G. F. (2004). Left hemisphere specialization for the control of voluntary movement rate. NeuroImage 22, 289–303. doi: 10.1016/j.neuroimage.2003.12.038, PMID: 15110019

[ref3] Annett M. (1978a). Genetic and nongenetic influences on handedness. Behav. Genet. 8, 227–249. doi: 10.1007/BF01072826, PMID: 687316

[ref4] Annett M. (1978b). Single gene explanation of right and left handedness and brainedness-the human handedness right shift factor behaves like a single gene. Coventry, England.

[ref5] Ashton G. C. (1982). Handedness: an alternative hypothesis. Behav. Genet. 12, 125–147. doi: 10.1007/BF01065761, PMID: 7126102

[ref6] Aune T. Aune M. Ettema G. Vereijken B. (2013). Comparison of bilateral force deficit in proximal and distal joints in upper extremities. Hum. Mov. Sci. 32, 436–444. doi: 10.1016/j.humov.2013.01.005, PMID: 23719626

[ref7] Aune T. K. Aune M. A. Ingvaldsen R. P. Vereijken B. (2017). Transfer of motor learning is more pronounced in proximal compared to distal effectors in upper extremities. Front. Psychol. 8:1530. doi: 10.3389/fpsyg.2017.01530, PMID: 28943857 PMC5596065

[ref8] Aune T. Ettema G. Vereijken B. (2016). Bilateral asymmetry in upper extremities is more pronounced in distal compared to proximal joints. J. Mot. Behav. 48, 143–152. doi: 10.1080/00222895.2015.1056766 26114377

[ref9] Baker S. N. (2011). The primate reticulospinal tract, hand function and functional recovery. J. Physiol. 589, 5603–5612. doi: 10.1113/jphysiol.2011.215160, PMID: 21878519 PMC3249036

[ref10] Besomi M. Hodges P. W. Clancy E. A. Van Dieën J. Hug F. Lowery M.. (2020). Consensus for experimental design in electromyography (CEDE) project: amplitude normalization matrix. J. Electromyogr. Kinesiol. 53:102438. doi: 10.1016/j.jelekin.2020.102438, PMID: 32569878

[ref11] Blanca-Mena M. J. Arnau J. García-Castro F. J. Alarcón-Postigo R. Bono Cabré R. (2022). Non-normal data in repeated measures ANOVA: impact on type I error and power. Psicothema 35, 21–29. doi: 10.7334/psicothema2022.292 36695847

[ref12] Brodal P. (2010). The central nervous system. Oxford, United Kingdom: Oxford University Press.

[ref13] Bruttini C. Esposti R. Bolzoni F. Cavallari P. (2016). Higher precision in pointing movements of the preferred vs. non-preferred hand is associated with an earlier occurrence of anticipatory postural adjustments. Front. Hum. Neurosci. 10:365. doi: 10.3389/fnhum.2016.00365, PMID: 27486394 PMC4947585

[ref14] Carpes F. P. Mota C. B. Faria I. E. (2010). On the bilateral asymmetry during running and cycling–a review considering leg preference. Phys. Ther. Sport 11, 136–142. doi: 10.1016/j.ptsp.2010.06.005, PMID: 21055708

[ref15] Cernacek J. (1961). Contralateral motor irradiation-cerebral dominance: its changes in hemiparesis. Arch. Neurol. 4, 165–172. doi: 10.1001/archneur.1961.00450080047005 13691977

[ref16] Changeux J.-P. Edelman G. M. (2017). The brain. New York: Routledge.

[ref17] Cheng J. Edwards L. J. Maldonado-Molina M. M. Komro K. A. Muller K. E. (2010). Real longitudinal data analysis for real people: building a good enough mixed model. Stat. Med. 29, 504–520. doi: 10.1002/sim.3775, PMID: 20013937 PMC2811235

[ref18] Cincotta M. Borgheresi A. Balzini L. Vannucchi L. Zeloni G. Ragazzoni A.. (2003). Separate ipsilateral and contralateral corticospinal projections in congenital mirror movements: neurophysiological evidence and significance for motor rehabilitation. Mov Disord 18, 1294–1300. doi: 10.1002/mds.10545, PMID: 14639670

[ref19] Cincotta M. Ziemann U. (2008). Neurophysiology of unimanual motor control and mirror movements. Clin. Neurophysiol. 119, 744–762. doi: 10.1016/j.clinph.2007.11.047, PMID: 18187362

[ref20] Cohen J. (2013). Statistical power analysis for the behavioral sciences. New York: Routledge.

[ref21] Corballis M. C. (1998). Cerebral asymmetry: motoring on. Trends Cogn. Sci. 2, 152–157. doi: 10.1016/s1364-6613(98)01156-5, PMID: 21227112

[ref22] Coren S. (1993). The lateral preference inventory for measurement of handedness, footedness, eyedness, and earedness: norms for young adults. Bull. Psychon. Soc. 31, 1–3. doi: 10.3758/BF03334122

[ref23] Coren S. Porac C. (1980). Family patterns in four dimensions of lateral preference. Behav. Genet. 10, 333–348. doi: 10.1007/BF01065596, PMID: 7213308

[ref24] Cratty B. J. (1962). Comparison of learning a fine motor task with learning a similar gross motor task, using kinesthetic cues. Res. Quarter. American Assoc. Health Physical Educ. Recreation 33, 212–221. doi: 10.1080/10671188.1962.10613193

[ref25] Čuk T. Leben-Seljak P. Štefančič M. (2001). Lateral asymmetry of human long bones. Poznań, Poland: Adam Mickiewicz University.

[ref26] de Boer B. J. Peper C. E. Beek P. J. (2013). Learning a new bimanual coordination pattern: interlimb interactions, attentional focus, and transfer. J. Mot. Behav. 45, 65–77. 23406196 10.1080/00222895.2012.744955

[ref27] de Poel H. J. Peper C. L. E. Beek P. J. (2007). Handedness-related asymmetry in coupling strength in bimanual coordination: furthering theory and evidence. Acta Psychol. 124, 209–237. 10.1016/j.actpsy.2006.03.003 16777042

[ref28] Delwaide P. Pepin J. (1991). The influence of contralateral primary afferents on Ia inhibitory interneurones in humans. J. Physiol. 439, 161–179. 1895236 10.1113/jphysiol.1991.sp018662 PMC1180104

[ref29] Farmer S. F. Bremner F. D. Halliday D. M. Rosenberg J. R. Stephens J. (1993). The frequency content of common synaptic inputs to motoneurones studied during voluntary isometric contraction in man. J. Physiol. 470, 127–155. 8308721 10.1113/jphysiol.1993.sp019851 PMC1143910

[ref30] Faul F. Erdfelder E. Lang A. G. Buchner A. (2007). G* power 3: a flexible statistical power analysis program for the social, behavioral, and biomedical sciences. Behav. Res. Methods 39, 175–191. doi: 10.3758/BF03193146, PMID: 17695343

[ref31] Gilbert A. N. Wysocki C. J. (1992). Hand preference and age in the United States. Neuropsychologia 30, 601–608. doi: 10.1016/0028-3932(92)90065-T, PMID: 1528408

[ref32] Gould H. r. Cusick C. Pons T. Kaas J. (1986). The relationship of corpus callosum connections to electrical stimulation maps of motor, supplementary motor, and the frontal eye fields in owl monkeys. J. Comp. Neurol. 247, 297–325. 3722441 10.1002/cne.902470303

[ref33] Green L. A. Gabriel D. A. (2018). The effect of unilateral training on contralateral limb strength in young, older, and patient populations: a meta-analysis of cross education. Phys. Ther. Rev. 23, 238–249. doi: 10.1080/10833196.2018.1499272

[ref34] Gueugneau N. Papaxanthis C. (2010). Time-of-day effects on the internal simulation of motor actions: psychophysical evidence from pointing movements with the dominant and non-dominant arm. Chronobiol. Int. 27, 620–639. doi: 10.3109/07420521003664205, PMID: 20524805

[ref35] Guiard Y. (1987). Asymmetric division of labor in human skilled bimanual action: the kinematic chain as a model. J. Mot. Behav. 19, 486–517. doi: 10.1080/00222895.1987.10735426, PMID: 15136274

[ref36] Hammond G. (2002). Correlates of human handedness in primary motor cortex: a review and hypothesis. Neurosci. Biobehav. Rev. 26, 285–292. doi: 10.1016/S0149-7634(02)00003-9, PMID: 12034131

[ref37] Harris L. J. (1991). The human infant in studies of lateralization of function: A historical perspective. A historical perspective. In Theory and Research in Behavioral Pediatrics. Eds. H. Fitzgerald, B. Lester and M. Yogman. vol. *5* (pp. 129–154). Boston, MA: Springer US.

[ref38] Heming E. A. Cross K. P. Takei T. Cook D. J. Scott S. H. (2019). Independent representations of ipsilateral and contralateral limbs in primary motor cortex. eLife 8:e48190. doi: 10.7554/eLife.48190, PMID: 31625506 PMC6824843

[ref1005] Hermens H. J. Freriks B. Disselhorst-Klug C. Rau G. (2000). Development of recommendations for SEMG sensors and sensor placement procedures. Journal of Electromyography and Kinesiology 10, 361–374. 11018445 10.1016/s1050-6411(00)00027-4

[ref39] Hortobágyi T. Taylor J. L. Petersen N. T. Russell G. Gandevia S. C. (2003). Changes in segmental and motor cortical output with contralateral muscle contractions and altered sensory inputs in humans. J. Neurophysiol. 90, 2451–2459. doi: 10.1152/jn.01001.2002, PMID: 14534271

[ref40] Huber R. (1999). Handwriting identification: Facts and fundamentals. Boca Raton, Florida: CRC press.

[ref41] Hugdahl K. (2005). Symmetry and asymmetry in the human brain. Eur. Rev. 13, 119–133. doi: 10.1017/S1062798705000700

[ref42] Jankowska E. Edgley S. Krutki P. Hammar I. (2005a). Functional differentiation and organization of feline midlumbar commissural interneurones. J. Physiol. 565, 645–658. doi: 10.1113/jphysiol.2005.083014, PMID: 15817636 PMC1464510

[ref43] Jankowska E. Krutki P. Matsuyama K. (2005b). Relative contribution of Ia inhibitory interneurones to inhibition of feline contralateral motoneurones evoked via commissural interneurones. J. Physiol. 568, 617–628. doi: 10.1113/jphysiol.2005.088351, PMID: 16096343 PMC1474749

[ref44] Jenny A. (1979). Commissural projections of the cortical hand motor area in monkeys. J. Comp. Neurol. 188, 137–145. doi: 10.1002/cne.901880111, PMID: 115906

[ref45] Johnson M. D. Thompson C. K. Tysseling V. M. Powers R. K. Heckman C. J. (2017). The potential for understanding the synaptic organization of human motor commands via the firing patterns of motoneurons. J. Neurophysiol. 118, 520–531. doi: 10.1152/jn.00018.2017, PMID: 28356467 PMC5511870

[ref46] Kiehn O. (2016). Decoding the organization of spinal circuits that control locomotion. Nat. Rev. Neurosci. 17, 224–238. doi: 10.1038/nrn.2016.9, PMID: 26935168 PMC4844028

[ref47] Konrad P. (Ed.) (2005). The abc of emg. In A practical introduction to kinesiological electromyography 1, 30–5. Arizona, USA.

[ref48] Kuypers H. G. (1964). The descending pathways to the spinal cord, their anatomy and function. In Progress in brain research, eds. J.C. Eccles and J.P. Scadé. vol. 11, 178–202. Cleveland, Ohio, USA. 10.1016/s0079-6123(08)64048-0 14300477

[ref49] Kuypers H. (1973). The anatomical organization of the descending pathways and their contributions to motor control especially in primates. In Human reflexes, pathophysiology of motor systems, Methodology of Human Reflexes, vol. 3. Basel: Karger Publishers, 38–68.

[ref50] Kuypers H. (1978). The motor system and the capacity to execute highly fractionated distal extremity movements. Electroencephalogr. Clin. Neurophysiol. Suppl. 34, 429–431. 108079

[ref52] Lecce E. Amoruso P. Vecchio A. D. Casolo A. Felici F. Farina D.. (2025a). Neural determinants of the increase in muscle strength and force steadiness of the untrained limb following a 4 week unilateral training. J. Physiol. 603, 3605–3630. doi: 10.1113/JP288954, PMID: 40500979

[ref53] Lecce E. Del Vecchio A. Nuccio S. Felici F. Bazzucchi I. (2025b). Higher dominant muscle strength is mediated by motor unit discharge rates and proportion of common synaptic inputs. Sci. Rep. 15:8269. doi: 10.1038/s41598-025-92737-8, PMID: 40065078 PMC11894131

[ref54] Liederman J. Kinsbourne M. (1980). The mechanism of neonatal rightward turning bias: a sensory or motor asymmetry? Infant Behav. Dev. 3, 223–238. doi: 10.1016/S0163-6383(80)80028-2

[ref55] Loffing F. Deeken O. Schorer J. (2024). Lateral preference in complex combat situations: prevalence and relationship with general measures of hand and foot preference. Laterality 29, 37–62. doi: 10.1080/1357650X.2023.2254004, PMID: 37671701

[ref56] Lorås H. Sigmundsson H. Talcott J. B. Öhberg F. Stensdotter A. (2012). Timing continuous or discontinuous movements across effectors specified by different pacing modalities and intervals. Exp. Brain Res. 220, 335–347. doi: 10.1007/s00221-012-3142-4, PMID: 22710620

[ref57] Matsumoto T. Watanabe T. Kuwabara T. Yunoki K. Chen X. Kubo N.. (2021). Excitability of the ipsilateral primary motor cortex during unilateral goal-directed movement. Front. Hum. Neurosci. 15:617146. doi: 10.3389/fnhum.2021.617146, PMID: 33679346 PMC7925409

[ref58] McManus I. C. (2002). Right hand, left hand: The origins of asymmetry in brains, bodies, atoms, and cultures. Cambridge, Massachusetts: Harvard University Press.

[ref59] Michel G. F. (2021). Handedness development: a model for investigating the development of hemispheric specialization and interhemispheric coordination. Symmetry 13:992. doi: 10.3390/sym13060992

[ref60] Moe-Nilssen R. Helbostad J. L. (2005). Interstride trunk acceleration variability but not step width variability can differentiate between fit and frail older adults. Gait Posture 21, 164–170. doi: 10.1016/j.gaitpost.2004.01.013, PMID: 15639395

[ref61] Mohr C. Thut G. Landis T. Brugger P. (2003). Hands, arms, and minds: interactions between posture and thought. J. Clin. Exp. Neuropsychol. 25, 1000–1010. doi: 10.1076/jcen.25.7.1000.16491, PMID: 13680446

[ref62] Nazmi N. Abdul Rahman M. A. Yamamoto S.-I. Ahmad S. A. Zamzuri H. Mazlan S. A. (2016). A review of classification techniques of EMG signals during isotonic and isometric contractions. Sensors 16:1304. doi: 10.3390/s16081304, PMID: 27548165 PMC5017469

[ref63] Oldfield R. (1971). Edinburgh handedness inventory. J. Abnorm. Psychol.

[ref64] Pandya D. N. Vignolo L. A. (1971). Intra-and interhemispheric projections of the precentral, premotor and arcuate areas in the rhesus monkey. Brain Res. 26, 217–233. doi: 10.1016/S0006-8993(71)80001-X 4993847

[ref65] Parma V. Brasselet R. Zoia S. Bulgheroni M. Castiello U. (2017). The origin of human handedness and its role in pre-birth motor control. Sci. Rep. 7:16804. doi: 10.1038/s41598-017-16827-y, PMID: 29196664 PMC5711880

[ref66] Peters M. Durding B. (1979). Left-handers and right-handers compared on a motor task. J. Mot. Behav. 11, 103–111. doi: 10.1080/00222895.1979.10735178, PMID: 15189803

[ref67] Petrovic I. Amiridis I. G. Kannas T. Tsampalaki Z. Holobar A. Sahinis C.. (2023). Footedness but not dominance influences force steadiness during isometric dorsiflexion in young men. J. Electromyogr. Kinesiol. 73:102828. doi: 10.1016/j.jelekin.2023.102828, PMID: 37782992

[ref68] Pierrot-Deseilligny E. Burke D. (2005). The circuitry of the human spinal cord: Its role in motor control and movement disorders. New York: Cambridge University Press.

[ref69] Porac C. Coren S. (1977). The assessment of motor control in sighting dominance using an illusion decrement procedure. Percept. Psychophys. 21, 341–346. doi: 10.3758/BF03199484

[ref70] Porac C. Coren S. (1981). Lateral preferences and human behavior. New York: Springer.

[ref71] Raymond M. Pontier D. (2004). Is there geographical variation in human handedness? Laterality 9, 35–51. doi: 10.1080/13576500244000274, PMID: 15382729

[ref72] Repp B. H. Penel A. (2004). Rhythmic movement is attracted more strongly to auditory than to visual rhythms. Psychol. Res. 68, 252–270. doi: 10.1007/s00426-003-0143-8, PMID: 12955504

[ref73] Rosenzweig E. S. Brock J. H. Culbertson M. D. Lu P. Moseanko R. Edgerton V. R.. (2009). Extensive spinal decussation and bilateral termination of cervical corticospinal projections in rhesus monkeys. J. Comp. Neurol. 513, 151–163. doi: 10.1002/cne.21940, PMID: 19125408 PMC2706096

[ref74] Rouiller E. M. Babalian A. Kazennikov O. Moret V. Yu X.-H. Wiesendanger M. (1994). Transcallosal connections of the distal forelimb representations of the primary and supplementary motor cortical areas in macaque monkeys. Exp. Brain Res. 102, 227–243. doi: 10.1007/BF00227511, PMID: 7705502

[ref75] Sahinis C. Amiridis I. G. Varvariotis N. Lykidis A. Kannas T. M. Negro F.. (2024). Foot-dominance does not influence force variability during ankle dorsiflexion and foot adduction. J. Sports Sci. 42, 1011–1021. doi: 10.1080/02640414.2024.2379699 39023311

[ref76] Šarabon N. Kozinc Ž. Löfler S. Hofer C. (2020). Resistance exercise, electrical muscle stimulation, and whole-body vibration in older adults: systematic review and meta-analysis of randomized controlled trials. J. Clin. Med. 9:2902. 32911822 10.3390/jcm9092902 PMC7563530

[ref77] Smoll F. L. Schutz R. W. (1978). Relationships among measures of preferred tempo and motor rhythm. Percept. Mot. Skills 46, 883–894. doi: 10.2466/pms.1978.46.3.883, PMID: 673648

[ref78] Swift E. J. (1903). Studies in the psychology and physiology of learning. Am. J. Psychol. 14, 201–251. doi: 10.2307/1412713

[ref79] Todor J. I. Kyprie P. M. (1980). Hand differences in the rate and variability of rapid tapping. J. Mot. Behav. 12, 57–62. doi: 10.1080/00222895.1980.10735205, PMID: 15215068

[ref80] Todor J. I. Kyprie P. M. Price H. L. (1982). Lateral asymmetries in arm, wrist and finger movements. Cortex 18, 515–523. doi: 10.1016/S0010-9452(82)80050-6, PMID: 7166039

[ref81] Truman G. Hammond G. R. (1990). Temporal regularity of tapping by the left and right hands in timed and untimed finger tapping. J. Mot. Behav. 22, 521–535. doi: 10.1080/00222895.1990.10735526, PMID: 15117660

[ref82] Uehara K. Morishita T. Kubota S. Hirano M. Funase K. (2014). P686: a comparison between short and long latency interhemispheric inhibition from the active to resting primary motor cortex during a unilateral muscle contraction. Clin. Neurophysiol. 125:S239.

[ref83] Vercauteren K. Pleysier T. Van Belle L. Swinnen S. P. Wenderoth N. (2008). Unimanual muscle activation increases interhemispheric inhibition from the active to the resting hemisphere. Neurosci. Lett. 445, 209–213. doi: 10.1016/j.neulet.2008.09.013, PMID: 18793696

[ref84] Zijdewind I. Butler J. E. Gandevia S. C. Taylor J. L. (2006). The origin of activity in the biceps brachii muscle during voluntary contractions of the contralateral elbow flexor muscles. Exp. Brain Res. 175, 526–535. doi: 10.1007/s00221-006-0570-z, PMID: 16924489

[ref85] Zijdewind I. Zwarts M. J. Kernell D. (1998). Influence of a voluntary fatigue test on the contralateral homologous muscle in humans? Neurosci. Lett. 253, 41–44. doi: 10.1016/S0304-3940(98)00609-0, PMID: 9754800

